# The Effect of Sacubitril-Valsartan on Ventricular Arrhythmia Burden in Patients With Heart Failure With Reduced Ejection Fraction

**DOI:** 10.7759/cureus.34508

**Published:** 2023-02-01

**Authors:** Paulo Medeiros, Cláudia Coelho, Cátia Costa-Oliveira, Sérgia Rocha

**Affiliations:** 1 Cardiology, Hospital de Braga, Braga, PRT; 2 Cardiology, University of Minho, Braga, PRT

**Keywords:** cardiac resynchronization therapy (crt), implantable cardiac defibrillator (icd), ventricular arrhythmia, heart failure with reduced ejection fraction, sacubitril-valsartan

## Abstract

Introduction

Heart failure with reduced ejection fraction (HFrEF) patients are prone to developing ventricular arrhythmias. In the PARADIGM-HF trial, sacubitril-valsartan (SV) showed a reduction in the composite endpoint of death and HF hospitalization in HFrEF patients; subgroup analysis of this trial revealed a reduction in both sudden death and deaths from worsening HF. The mechanism by which SV may affect the incidence of ventricular arrhythmias is currently under debate, and the literature provides conflicting results. The aim of our study was to evaluate the potential antiarrhythmic effect of this drug in patients with HFrEF carrying an implantable cardiac defibrillator (ICD) or a cardiac resynchronization therapy with a defibrillator (CRT-D).

Methods

This was a single-center, observational and retrospective study. Inclusion criteria were implantation of an ICD or CRT-D device between 2009 and 2019, age ≥18 years, left ventricle ejection fraction (LVEF) ≤40%, New York Heart Association (NYHA) functional class ≥II, and treatment with an angiotensin-converting enzyme inhibitor or an angiotensin receptor blocker for at least 12 months, followed by replacement with SV. Exclusion criteria were NYHA class IV, frequent alterations in chronic medication for HFrEF, and implantation of an ICD or CRT-D after the introduction of SV. The primary outcome was the occurrence of ventricular arrhythmias in the form of appropriate device shocks, ventricular fibrillation, or ventricular tachycardia. The comparisons were performed between two periods of time (12 months before and 12 months after SV) in the same group of patients.

Results

Fifty-four patients met the inclusion criteria. The mean age was 69.5 ± 1.65 years, and 74.1% of patients were male. The number of patients experiencing appropriate shocks was significantly lower after SV initiation (2% vs. 18%; p=0.016). The percentage of VT (13 vs. 20%; p=0.549) and VF episodes (4% vs. 13% for VF; p=0.289) were also lower, but these differences were not statistically significant. There were no significant differences in the value of NT-proBNP (1128 vs. 775 pg/mL; p=0.858), LVEF (28.4 vs. 29.6%; p=0.315), and left ventricular end-diastolic diameter (65.0 vs. 66.0 mm; p=0.5492).

Conclusion

SV seems to reduce the risk of arrhythmic events requiring appropriate shock therapy.

## Introduction

Heart failure (HF) is a clinical syndrome characterized by cardinal symptoms (with or without signs) secondary to structural or functional abnormalities of the heart, resulting in increased intracardiac pressures and/or inadequate output [1]. HF has a significant impact on quality of life and prognosis, especially in heart failure with reduced ejection fraction (HFrEF), in which the left ventricle ejection fraction (LVEF) is <40% [2]. Patients with HF are prone to developing ventricular arrhythmias [3]. Sudden death is responsible for a significant proportion of deaths [4]. However, its incidence seems to be decreasing, most likely due to the advance in HFrEF treatment options, including pharmacological therapies and implantable cardiac devices. Indeed, LVEF is the key factor when considering an implantable cardioverter-defibrillator (ICD) in the primary prevention of sudden death [1]. Although defibrillators may prevent sudden death, these devices do not reduce the incidence of ventricular arrhythmias [5]. Sacubitril-Valsartan (SV) is an angiotensin receptor-neprilysin inhibitor (ARNI) that showed a reduction in the composite endpoint of death and HF hospitalization in HFrEF patients, compared to enalapril [6]. It is currently one of the first-line drugs in this population. The precise mechanism by which SV reduces mortality is not fully understood. However, a subgroup analysis of the PARADIGM-HF trial revealed a reduction in both sudden death and deaths from worsening HF [7]. Cardiac remodeling following chronic neurohormonal activation may provide the necessary substrate for ventricular arrhythmias and, consequently, sudden death [8]. Although small studies showed conflicting results, it has been suggested that SV may display antiarrhythmic properties. The mechanism by which SV may affect the incidence of ventricular arrhythmias is currently under debate; possible explanations include reverse remodeling with LVEF recovery or a direct effect on ventricular conduction properties. The aim of our study was to evaluate the potential antiarrhythmic effect of SV in patients with HFrEF carrying an ICD or cardiac resynchronization therapy with a defibrillator (CRT-D).

## Materials and methods

Study design and population

This was a single-center, observational, retrospective, and longitudinal study. It complied with the Declaration of Helsinki and was conducted in accordance with the local ethics committee requirements. Patients were included based on the following criteria: (1) implantation of an ICD or CRT-D device between 2009 and 2019, (2) age ≥18 years, (3) LVEF ≤40%, (4) New York Heart Association (NYHA) functional class ≥II and (5) treatment with an angiotensin-converting enzyme inhibitor (ACEi) or an angiotensin receptor blocker (ARB) for at least 12 months, followed by replacement with SV. Exclusion criteria were (1) NYHA functional class IV, (2) frequent alterations in chronic medication for HFrEF (defined as introduction or removal of at least two HFrEF disease modifying drugs during the 12 months prior to SV or HFrEF drug dose up or down-titration at least three times during the 12 months prior to SV, except for diuretics), preventing the identification of “steady state” pharmacological therapy and (3) implantation of an ICD or CRT-D after the introduction of SV. 

Data and definitions

Clinical, biochemical, and echocardiographic data were retrospectively collected. Cardiovascular risk factors included hypertension (systolic blood pressure of ≥140 mmHg, diastolic blood pressure of ≥90 mmHg or use of antihypertensive medication), dyslipidemia (total cholesterol ≥200 mg/dL or use of lipid-lowering medication), diabetes mellitus (hemoglobin A1c levels of ≥6.5% or use of an antidiabetic treatment) and smoking (considered only if the patient was an active smoker). Other relevant clinical features were chronic medication (including BB, ACEi, ARB, antiarrhythmic drugs, and diuretics), previous history of coronary artery disease (epicardial coronary stenosis >50% in the left main artery or >70% in other relevant vessels) and chronic kidney disease (glomerular filtration rate (GFR) <60 mL/min). Biochemical and echocardiographic data were collected before and after SV initiation. The considered variables were GFR (calculated using the Cockroft-Gault formula), NT-proBNP, LVEF, and left ventricle end-diastolic diameter (LVEDD). Data regarding the occurrence of ventricular arrhythmias (including sustained and non-sustained ventricular tachycardia and ventricular fibrillation) were obtained by interrogating each implantable cardiac device. The appropriate shock was defined as therapy triggered by sustained VT or VF. The inappropriate shock was defined as therapy triggered by supraventricular tachycardia, signal oversensing, or device malfunction.

Primary and secondary outcomes

The primary outcome of this study was the occurrence of ventricular arrhythmias in the form of appropriate device shocks, ventricular fibrillation (VF), or ventricular tachycardia (VT). Secondary outcomes were changes in GFR, NT-proBNP, LVEF, and LVEDD.

Statistical analysis

The final database was divided into two separate periods: 12 months before the introduction of SV and 12 months after the introduction of the drug. The comparisons were performed between these two periods of time in the same group of patients. Categorical variables were expressed as absolute (n) and relative (%) frequency. Quantitative variables with normal distribution were expressed as the mean ± SD; those with non-normal distribution were presented as median and interquartile ranges. The student’s t-test (for normally distributed variables) or the Wilcoxon rank-sum test (for non-parametrically distributed variables) were used to compare groups. Categorical variables were compared using McNemar’s test. For Windows, statistical analysis was performed using SPSS (version 27 IBM Corp, Armonk, NY). A p-value <0.05 was considered statistically significant, and only two-sided tests were conducted.

## Results

Baseline characteristics

**Figure 1:**
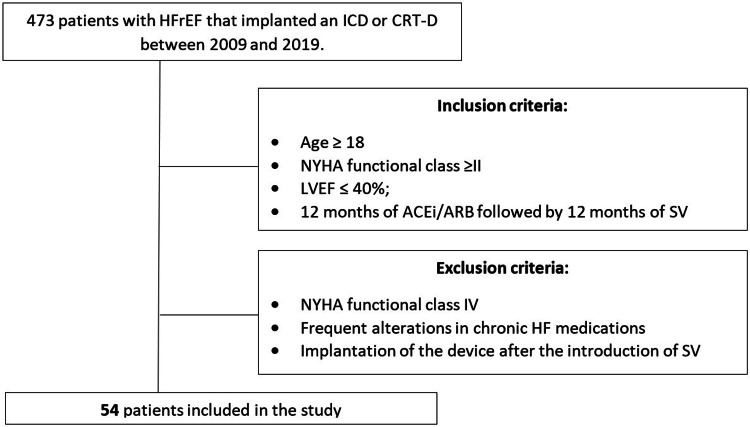
Patient selection flowchart. ACEi: Angiotensin-converting enzyme inhibitor; ARB: Angiotensin receptor blocker; CRT-D: Cardiac resynchronization therapy with defibrillator; HF: Heart failure; HFrEF: Heart failure with reduced ejection fraction; ICD: Implantable cardioverter-defibrillator; LVEF: Left ventricle ejection fraction; NYHA: New York Heart Association; SV: Sacubitril-Valsartan.

We identified 473 patients with HFrEF who implanted an ICD or CRT-D in our center between 2009 and 2019. After applying inclusion and exclusion criteria, 54 patients were included in the study (Figure 1).

The mean age of the population was 69.5 ± 1.7 years, and 74.1% of patients were male (n=40). Mean LVEF before the introduction of the ARNI was 28.4% ± 1.4, and all the patients were under treatment with ACEi/ARB and BB. The coronary disease was present in 59.6% of cases (n=32). Baseline characteristics are detailed in Table 1.

**Table 1:** Baseline characteristics of the population. ACEi: Angiotensin-converting enzyme inhibitor; ARB: Angiotensin receptor blocker; BB: Beta-blocker; CRT-D: Cardiac resynchronization therapy with defibrillator; GFR: Glomerular filtration rate; IQR: Interquartile range; ICD: Implantable cardioverter-defibrillator; LVEDD: Left ventricle end-diastolic diameter; LVEF: Left ventricle ejection fraction; MRA: Mineralocorticoid-receptor antagonist.

Characteristics	
Male gender, n (%)	40 (74.1)
Mean age, years ± SD	69.5±1.7
Hypertension, n (%)	45 (83.3)
Dyslipidemia, n (%)	29 (53.7)
Diabetes mellitus, n (%)	38 (70.4)
Smoking, n (%)	18 (33.3)
Coronary artery disease, n (%)	32 (59.6)
Chronic kidney disease, n (%)	17 (31.5)
Treatment with BB, n (%)	54 (100)
Treatment with ACEi/ARB, n (%)	54 (100)
Treatment with MRA, n (%)	39 (72.2)
Treatment with Amiodarone, n (%)	12 (22.2)
Mean GFR, mL/min ± SD	69.1±3.8
Median NT-proBNP, pg/mL (IQR)	1128 (3137)
Mean LVEF, % ± SD	28.4±1.4
Median LVEDD, mm (IQR)	65.0 (6.1)
ICD, n (%)	30 (55.6)
CRT-D, n (%)	24 (44.4)
Primary prevention of sudden death	46 (85.2)

Primary outcomes

The number of patients experiencing appropriate shocks was significantly lower after ARNI initiation compared to the 12 months of ACEi/ARB (2% vs. 18%; p=0.016). The percentage of VT (13 vs. 20%; p=0.549) and VF episodes (4% vs. 13% for VF; p=0.289) among the studied population was also lower after the introduction of SV, but these differences were not statistically significant (Table 2).

**Table 2:** Primary outcomes. ^1 ^McNemar test. ARNI: Angiotensin receptor-neprilysin inhibitor; VT: Ventricular tachycardia.

Event	12 months prior to ARNI	12 months after ARNI	P-value	Effect size (Cohen G)
Appropriate device shock, n (%)	8 (18)	1 (2)	0.0161	0.5
Ventricular fibrillation, n (%)	6 (13)	2 (4)	0.2891	0.25
Sustained VT, n (%)	9 (20)	6 (13)	0.5491	0.15
Non-sustained VT, n (%)	14 (31)	11 (25)	0.5491	0.15

Secondary outcomes

Regarding biochemical parameters, the calculated GFR using the Cockroft-Gault formula was slightly lower 12 months after the initiation of SV, reaching statistical significance (69.1 vs. 63.8 mL/min; p=0.028). The median value of NT-proBNP was not significantly different before and after ARNI. None of the evaluated echocardiographic measures have been found to be statistically different in the compared periods (Table 3).

**Table 3:** Secondary outcomes. ^2^ Student's t-test; ^3^ Wilcoxon rank-sum test. ARNI: Angiotensin receptor-neprilysin inhibitor; GFR: Glomerular filtration rate; IQR: Interquartile range; LVEDD: Left ventricle end-diastolic diameter; LVEF: Left ventricle ejection fraction.

	12 months prior to ARNI	12 months after ARNI	P-value	Effect Size (Cohen G)
GFR, mean mL/min (SD)	69.1 (3.82)	63.8 (3.7)	0.0282	1.39
NT-proBNP, median pg/mL (IQR)	1128 (3137)	775 (1575)	0.8583	-0.03
LVEF, mean % (SD)	28.4 (1.4)	29.6 (1.2)	0.3152	0.92
LVEDD, median mm (IQR)	65.0 (6.1)	66.0 (8.0)	0.5493	-0.07

## Discussion

The main point of our results is that SV reduces the risk of arrhythmic events requiring appropriate shock therapy, even without a significant change in echocardiographic LV remodeling parameters. As anticipated by the inclusion criteria, our HFrEF population included high-risk patients with low LVEF that were referred to the implantation of a defibrillator device. As previously stated, the literature provides conflicting results regarding the effect of this drug on the incidence of ventricular arrhythmias. A prospective study by de Diego C et al. [9] revealed a significant reduction in appropriate ICD shocks and ventricular arrhythmias in-home monitoring of ICD patients after ARNI. With a similar design to the current study, Martens P et al. [10] evaluated the same group of patients, comparing pre and post-ARNI periods; the investigators found that SV was associated with a lower degree of VT/VF and, consequently, less ICD therapies, but unlike our study, this effect was parallel to an improvement in cardiac reverse remodeling parameters. On the other hand, a retrospective study by El-Battrawy I et al. [11] concluded that SV does not reduce the risk of ventricular arrhythmias in patients with HFrEF over 12 months of follow-up, despite a significant improvement in LVEF. These different results suggest that there may be more than one pathway involved in modulating the arrhythmic risk in patients treated with ARNI.

LV reverse remodeling is a common goal of neurohormonal antagonists and is associated with improved prognosis [12]. Besides being proved for older drug classes such as ACEi or BB, the ability to induce reverse remodeling is also well described for ARNI; indeed, multiple studies revealed that SV is associated with improved LVEF, reduced atrial and ventricular volumes, lower natriuretic peptide values and improved diastolic function [13-15]. Considering that elevated levels of NT-proBNP and elevated filling pressures are independent predictors of sustained VT and appropriate device therapies [16,17], it is intuitive to regard LV reverse remodeling as a plausible mechanism for reducing ventricular arrhythmias in the HFrEF population. However, as previously described, some studies report improvement in LVEF without decreasing the incidence of ventricular arrhythmias; conversely, our study showed that the number of events requiring appropriate shock decreased, despite the non-significant decrease in sustained VT or VT and the non-significant improvement in LVEF or LVEDD. This may suggest that the reduction of ventricular arrhythmias is only partially explained by reverse remodeling, and other mechanisms may be considered [18]. Other hypothesized mechanisms may be future investigation targets, such as the sympatholytic effect of neprilysin pathway inhibition. Finally, our study identified a numerically small but statistically significant decrease in mean GFR. This effect has been previously associated with SV treatment [19] but may also be a marker of HFrEF progression. Our study has some important limitations that should be stated. First, it is a retrospective study based on computer records, which intrinsically prevents the control of possible confounding variables. Second, due to the single-center nature of the study, the application of inclusion and exclusion criteria resulted in a relatively small sample of patients. Also, although the study has the advantage of analyzing the same population in different periods of time, there was no control group to parallel the results. Despite the information that each patient was taking the highest tolerated dose of SV, the exact dose was missing from some medical registries. Finally, even though our laboratory has standardized practices regarding echocardiographic measures, there is operator dependence, which may affect the uniformity of the collected parameters.

## Conclusions

SV reduces the risk of arrhythmic events requiring appropriate shock therapy. The number of patients experiencing appropriate shocks was significantly lower after ARNI; a reduction was also obtained regarding VF and sustained VT, but this difference was not statistically significant. This effect was observed without a concomitant improvement in reverse remodeling parameters, as provided by the secondary outcomes of LVEF and LVEDD. Finally, there was a small but significant reduction in mean GFR, reaching statistical significance, and a decrease in median NT-proBNP value, although the latter was not statistically significant. Larger and prospective studies are warranted to further clarify the potential antiarrhythmic properties of SV and the involved biological mechanism.

## References

[1] McDonagh TA, Metra M, Adamo M (2021). 2021 ESC Guidelines for the diagnosis and treatment of acute and chronic heart failure. Eur Heart J.

[2] Pocock SJ, Ariti CA, McMurray JJ (2013). Predicting survival in heart failure: a risk score based on 39 372 patients from 30 studies. Eur Heart J.

[3] Phillips PA, Burrell LM, Tonkin AM, Johnston CI (1995). Congestive cardiac failure and arrhythmias. Med J Aust.

[4] Cosmi D, Mariottoni B, Cosmi F (2017). Declining risk of sudden death in heart failure. N Engl J Med.

[5] Zeppenfeld K, Tfelt-Hansen J, de Riva M (2022). 2022 ESC Guidelines for the management of patients with ventricular arrhythmias and the prevention of sudden cardiac death. Eur Heart J.

[6] Timmis A (2014). Neprilysin inhibition for heart failure. N Engl J Med.

[7] Desai AS, McMurray JJ, Packer M (2015). Effect of the angiotensin-receptor-neprilysin inhibitor LCZ696 compared with enalapril on mode of death in heart failure patients. Eur Heart J.

[8] Packer M (2020). What causes sudden death in patients with chronic heart failure and a reduced ejection fraction?. Eur Heart J.

[9] de Diego C, González-Torres L, Núñez JM (2018). Effects of angiotensin-neprilysin inhibition compared to angiotensin inhibition on ventricular arrhythmias in reduced ejection fraction patients under continuous remote monitoring of implantable defibrillator devices. Heart Rhythm.

[10] Martens P, Lambeets S, Lau CW, Dupont M, Mullens W (2019). Impact of sacubitril/valsartan on heart failure admissions: insights from real-world patient prescriptions. Acta Cardiol.

[11] El-Battrawy I, Pilsinger C, Liebe V (2019). Impact of sacubitril/valsartan on the long-term incidence of ventricular arrhythmias in chronic heart failure patients. J Clin Med.

[12] Reis Filho JR, Cardoso JN, Cardoso CM, Pereira-Barretto AC (2015). Reverse cardiac remodeling: a marker of better prognosis in heart failure. Arq Bras Cardiol.

[13] Januzzi JL Jr, Prescott MF, Butler J (2019). Association of change in N-terminal pro-B-type natriuretic peptide following initiation of sacubitril-valsartan treatment with cardiac structure and function in patients with heart failure with reduced ejection fraction. JAMA.

[14] Desai AS, Solomon SD, Shah AM (2019). Effect of sacubitril-valsartan vs enalapril on aortic stiffness in patients with heart failure and reduced ejection fraction: a randomized clinical trial. JAMA.

[15] Wang Y, Zhou R, Lu C, Chen Q, Xu T, Li D (2019). Effects of the angiotensin-receptor neprilysin inhibitor on cardiac reverse remodeling: meta-analysis. J Am Heart Assoc.

[16] Alvarez PA, Truong CN, Briasoulis A, Ganduglia-Cazaban C (2019). Prescription of potentially harmful drugs in young adults with heart failure and reduced ejection fraction. Am J Cardiol.

[17] Seegers J, Bergau L, Expósito PM (2016). Prediction of appropriate shocks using 24-hour Holter variables and T-wave alternans after first implantable cardioverter-defibrillator implantation in patients with ischemic or nonischemic cardiomyopathy. Am J Cardiol.

[18] Vecchi AL, Abete R, Marazzato J, Iacovoni A, Mortara A, De Ponti R, Senni M (2022). Ventricular arrhythmias and ARNI: is it time to reappraise their management in the light of new evidence?. Heart Fail Rev.

[19] Hubers SA, Brown NJ (2016). Combined angiotensin receptor antagonism and neprilysin inhibition. Circulation.

